# HTLV-1 infection and associated diseases in Peruvian twins probably exposed to HTLV-1 mother-to-child transmission

**DOI:** 10.1186/1742-4690-8-S1-A57

**Published:** 2011-06-06

**Authors:** Carolina Alvarez, Elsa Gonzalez, Kristien Verdonck, Eduardo Gotuzzo

**Affiliations:** 1 Instituto de Medicina Tropical Alexander von Humboldt, Universidad Peruana Cayetano Heredia, Lima, Perú; 2 Facultad de Medicina, Universidad Peruana Cayetano Heredia, Lima, Perú; 3 Institute of Tropical Medicine, Antwerp, Belgium; 4 Departamento de Enfermedades Infecciosas, Tropicales y Dermatología, Hospital Nacional Cayetano Heredia, Lima, Perú

## Introduction

Since 1989, our Institute provides HTLV-1 screening to the relatives of newly diagnosed HTLV-1 cases.

## Methods

We report HTLV-1 infection and associated diseases in twin pairs in which both siblings were tested at ages older than 3.

## Results

We identified six pairs of twins. Among four monozygotic pairs, 3 were male; median age at HTLV-1 screening was 19. Two pairs were HTLV-1-negative, one positive, and one discordant. The mother of the concordant positive pair was tested 32 years after twins’ delivery because of strongyloidiasis, and developed Adult-T-cell- leukemia-lymphoma (ATLL). Length of breastfeeding was reported the same for both twins, but not recalled. At age 35 one developed ATLL, the other remained asymptomatic. In the discordant monozygotic pair, length of breastfeeding was not clarified for the negative sibling, and six months for the HTLV-1 positive one; the latter developed crusted scabies at age 15. In the concordant negative pairs, born to mothers who later developed HAM/TSP, each twin was breastfed for less than three months. In the case of dizygotic twins, four female, both pairs were HTLV-1-discordant. In one pair, whose mother was HTLV-1-positive, twins were equally breastfed for six months. In the other pair, only the positive sibling received breastfeeding, for two years. Both positive siblings presented HAM/TSP. According to medical interviews, no other risk factors for HTLV-1-adult transmission were present in these twins.

## Conclusions

These findings suggest the interplay of genetic and non genetic factors, including the confirmed role of breastfeeding, on HTLV-1 mother-to-child transmission and HTLV-1-associated diseases.

